# Psychometric Properties and Network Structure of the Domain-Specific Climate Change Distress Scale

**DOI:** 10.1177/24705470261469261

**Published:** 2026-07-18

**Authors:** Martin Weiß, Julian Gutzeit

**Affiliations:** 1 Department of Psychology I: Clinical Psychology and Psychotherapy, Institute of Psychology, 9190 University of Würzburg, Würzburg, Germany; 2 Department of Psychology I: Psychotherapy and Intervention Psychology, Institute of Psychology, 9190 University of Würzburg, Würzburg, Germany

**Keywords:** climate distress, eco-anxiety, network analysis, measurement invariance, psychometrics

## Abstract

**Background:**

Climate change increasingly poses a threat to psychological health. The Domain-Specific Climate Change Distress Scale (DCCDS) was developed to capture climate-related distress across six thematic (ecology, existence, food supply, future generations, society, and wealth) and one generic domain. Building on its theoretical foundation, the present study extends initial validation efforts by providing large-scale psychometric data and normative values, drawing on a large, diverse sample to further substantiate the scale’s psychometric properties and practical utility.

**Methods:**

We pooled data from seven independent German-speaking samples (N = 894; Mage = 39.2, SD = 13.5; 53% women). We replicated the bifactor S-1 model using confirmatory factor analysis (CFA), tested measurement invariance across gender, and estimated a regularized network. Exploratory graph analysis (EGA) served as a dimensionality check. Bootstrap stability analyses and a network comparison test (NCT) assessed the robustness of network indices and gender differences.

**Results:**

CFA confirmed acceptable fit of the bifactor S-1 model. Metric invariance across gender was fully supported; partial scalar invariance was established after freeing intercepts for three items. In the regularized network, items related to food supply, future generations, and generic climate anxiety showed the highest centrality. EGA identified six stable empirical dimensions, with society and wealth items loading onto a single community. NCT revealed equivalent global network structure across gender. Gender-stratified and age-stratified normative percentiles are provided.

**Conclusion:**

The DCCDS demonstrates robust psychometric properties across independent samples and is largely measurement-equivalent across gender. Findings support its use for individual diagnostics and group comparisons in climate psychology research and clinical settings. The empirical identification of six rather than seven dimensions further invites reconsideration of the Society and Wealth domain distinction. Normative data facilitate score interpretation in applied settings.

## Introduction

Climate change has emerged as one of the defining psychological challenges of the 21st century. Growing empirical evidence documents that exposure to climate-related threats and the anticipation of future ecological collapse can give rise to clinically relevant distress responses, broadly captured under the constructs of eco-anxiety, climate grief, and climate-related distress.^1,2^ While these phenomena are increasingly recognized in the clinical literature, their systematic measurement remains a critical bottleneck for research and practice alike. Existing scales tend either to focus exclusively on pathological levels of climate anxiety - thus neglecting subclinical manifestations - or to assess only its general dimensions, failing to differentiate the specific aspects associated with the heterogeneous consequences of the climate crisis (e.g., Refs. 3 and 4).

The Domain-Specific Climate Change Distress Scale (DCCDS; Ref. 5) was developed to address this gap by providing a multidimensional measure of climate-related distress. Building on a bifactor modelling approach, the DCCDS captures both a strong general factor of climate distress and six domain-specific facets: ecology, existence, food supply, future generations, society, and wealth. The bifactor S-1 structure - in which the generic subscale serves as the reference factor loading exclusively on the general factor, while all other subscales additionally carry specific factor variance - reflects the theoretical assumption that climate distress is simultaneously unitarily experienced and domain-differentiated.^6^

To date, the DCCDS has been validated using traditional methods of scale development, but a comprehensive psychometric characterization drawing on pooled, multi-study data has not been conducted. Such analyses are essential for three reasons. First, adequate statistical power for complex network analyses typically requires samples exceeding 500 participants.^7^ Second, the clinical utility of a scale depends critically on measurement invariance across relevant subgroups, particularly gender, given documented gender differences in climate anxiety.^3^ Third, clinically useful normative benchmarks require representative, sufficiently large reference samples.

Network psychometrics offers a complementary framework to traditional factor-analytic approaches. Rather than representing psychological constructs as latent common causes of observed item responses, network models characterize constructs as systems of mutually reinforcing item associations.^8,9^ This perspective is particularly informative for heterogeneous constructs such as climate distress, where domain-specific pathways of item activation may carry distinct psychological meaning.

The present study pursued four aims: (1) to replicate the bifactor S-1 structure of the DCCDS in the largest sample assembled to date; (2) to assess measurement invariance across gender; (3) to characterize the network topology of DCCDS items, including centrality indices and bridge symptoms; and (4) to provide gender- and age-stratified normative data for practical use.

## Methods

### Participants and Procedure

Data were pooled from seven independent German-speaking samples collected between June 2023 and February 2026. After excluding participants who did not respond positively to a dichotomous self-reported proficiency item to assess careless responding and removing duplicate cases across studies, the individual sample sizes were as follows: *N* = 305 (June 2023), *N* = 78 (July 2023), *N* = 105 (November 2023), *N* = 145 (June 2024), *N* = 128 (July 2024), *N* = 71 (June 2025), and *N* = 62 (February 2026) yielding a final analytic sample of N = 894 participants with complete DCCDS responses (M_age_ = 39.2 years, SD = 13.5, range 18–76; 53% women, 46% men, 1.0% other/not specified). A gender-by-age-group cross-tabulation and sample-wise demographic summaries are provided in the Supplementary Material (Tables S3-S5). Subjective social status, assessed with the MacArthur Scale of Subjective Social Status, averaged *M* = 5.43 (*SD* = 1.62). The sample was comparatively highly educated (66.4% reported a university entrance qualification (Abitur) or a university degree); and self-reported net monthly income, assessed in six of the seven subsamples (*n* = 749), was broadly distributed, most frequently falling in the €1,000–€1,500 category. Full education and income distributions are reported in Supplementary Table S5.

### Measure

The DCCDS consists of 28 items assessing climate-related psychological distress across seven domains of four items each: Generic distress, Ecology, Existence, Food supply, Future generations, Society, and Wealth. Items are rated on a 7-point Likert scale (1 = not at all distressed to 7 = extremely distressed). All items of DCCDS can be found on Open Science Framework (OSF; https://doi.org/10.17605/OSF.IO/4QMG5). The bifactor S-1 model specifies the Generic subscale as the reference factor (loadings on this generic factor only), with the remaining six subscales each additionally carrying a domain-specific factor orthogonal to both the generic factor and all other specific factors.^10^

### Statistical Analyses

All analyses were conducted in R (version 4.6.0). Mean scores for each subdomain were compared across samples using one-way ANOVAs; domain-level *p*-values were corrected using the Benjamini–Hochberg FDR procedure. Gender differences in normative scores were examined using independent-samples tests with Cohen’s d as effect size. Age-related patterns were evaluated using linear and quadratic regression models, with model improvement assessed by ΔR^2^ and nested *F*-tests. Confirmatory factor analysis was performed using the *lavaan* package^11^ with robust maximum likelihood estimation (MLR). Model fit was evaluated using standard criteria: CFI/TLI > .90 (acceptable) and > .95 (good), RMSEA < .08 (acceptable), SRMR < .08.^12^ To complement the factor-analytic results, model-based reliability coefficients (omega total and omega-hierarchical-subscale) were computed from the standardized bifactor S-1 loadings. Measurement invariance across gender was tested sequentially (configural, metric, scalar) and evaluated using ΔCFI < −.010 and ΔRMSEA < +.015 as thresholds.^13^ Items violating scalar invariance were identified via the *lavTestScore* function and freed to establish partial scalar invariance.^14^

The regularized network was estimated using EBIC-GLASSO (γ = 0.5) with polychoric correlations via the *bootnet* package.^15^ Because the strong generic factor produced a dense regularized solution, we additionally estimated a residual network by partialling out generic factor scores prior to GLASSO estimation. Item centrality (Strength, Betweenness, Closeness) and bridge symptoms were computed using *qgraph*^16^ and *networktools*. Stability was assessed via case-dropping bootstrap (nBoots = 1,000) and the CS-coefficient.^7^ Exploratory graph analysis (EGA) with the walktrap community detection algorithm and 500 bootstrap replications was performed using *EGAnet*.^17^ Gender differences in network structure were tested with the Network Comparison Test (NCT; Ref. 18). Edge-level NCT tests were corrected for multiple comparisons using the Benjamini–Hochberg false discovery rate procedure.

## Results

### Descriptive Statistics

Item means ranged from 3.61 to 5.40, indicating that all items – except one from the existence domain and one from the wealth domain-were scored above the scale midpoint on average, with considerable variation in scores across items (see Supplementary Table S1 for full item-level descriptive statistics). Between-sample heterogeneity was small. Subscale-specific ANOVAs indicated statistically significant but small differences for Generic, *F*(6, 887) = 2.63, *p* = .016, η^2^ = .018, *p*_FDR_ = .027; Future, *F*(6, 887) = 3.94, *p* = .001, η^2^ = .026, *p*_FDR_ = .005; Society, *F*(6, 887) = 3.09, *p* = .005, η^2^ = .021, *p*_FDR_ = .014; and Wealth, *F*(6, 887) = 3.06, *p* = .006, η^2^ = .020, *p*_FDR_ = .014. Ecology reached significance before correction, *F*(6, 887) = 2.15, *p* = .046, η^2^ = .014, but not after FDR correction, *p*_FDR_ = .064. Existence, *F*(6, 887) = 0.52, *p* = .792, η^2^ = .004, *p*_FDR_ = .792, and Food, *F*(6, 887) = 1.30, *p* = .253, η^2^ = .009, *p*_FDR_ = .295, showed no significant sample effects. Thus, pooling was considered defensible, while retaining a transparent record of modest mean-level heterogeneity across samples.

### Confirmatory Factor Analysis

The bifactor S-1 model showed acceptable fit: χ^2^(326) = 1663.07, CFI = .918, TLI = .904, RMSEA = .079 [90% CI: .075, .083], SRMR = .052. All factor loadings on the general factor were statistically significant (ps < .001), ranging from λ = .544 (WEALTH3) to λ = .841 (GENERIC4). Specific factor loadings were similarly significant across all domains, with the exception of FOOD1 (λ = .167) and FOOD2 (λ = .194), whose variance was predominantly accounted for by the general factor (for factor loadings, see Table S2 in the Supplementary Material). Model-based reliability estimates derived from the bifactor S-1 solution are reported in Table S6.

### Measurement Invariance

Sequential tests of measurement invariance across gender are presented in Table 1. Configural and metric invariance were well-supported, with negligible change in CFI between models (ΔCFI = .000). Full scalar invariance was statistically rejected (Δχ^2^(21) = 50.31, p < .001), though the associated ΔCFI of −.001 fell well below the recommended threshold of −.010. Score tests identified three non-invariant intercepts: FOOD1, EXISTENCE1, and SOCIETY1. Freeing these intercepts yielded a partial scalar model that did not differ significantly from the metric model (Δχ^2^(18) = 14.93, p = .667), confirming partial scalar invariance. Each of the six specific factors and the general factor retained at least three invariant intercepts after freeing, satisfying the minimum condition for meaningful latent mean comparison.^14^

**Table 1.** Measurement Invariance Tests Across Gender (N = 885, Male and Female Gender Only)

Model	χ^2^(df)	CFI	TLI	RMSEA [90% CI]	SRMR	Δχ^2^ p
Configural	2674.0 (652)	.913	.899	.079 [.076, .083]	.053	-
Metric	2727.7 (697)	.913	.906	.077 [.073, .081]	.057	.40
Scalar	2778.0 (718)	.912	.907	.076 [.073, .080]	.058	< .001
Partial scalar	2742.8 (715)	.913	.908	.076 [.072, .080]	.057	.67

*Note.* MLR estimation. ΔCFI and ΔRMSEA relative to the preceding model. Partial scalar model frees intercepts for FOOD1, EXISTENCE1, and SOCIETY1.

### Network Analysis

**Figure 1.**
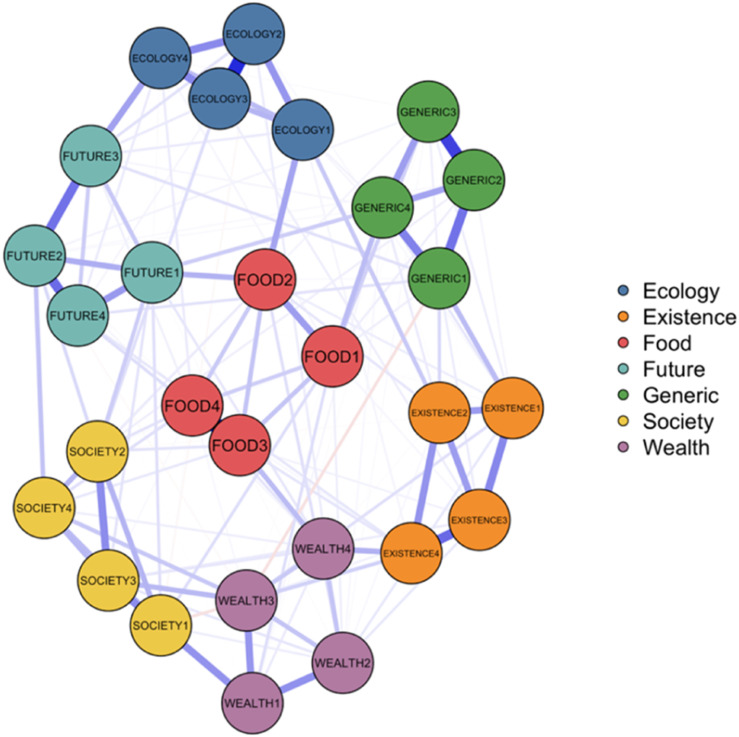
EBIC-GLASSO regularized network of DCCDS items. Node colors indicate theoretical domain membership. Edge thickness represents partial correlation strength; blue edges indicate positive, red edges negative associations

**Figure 2.**
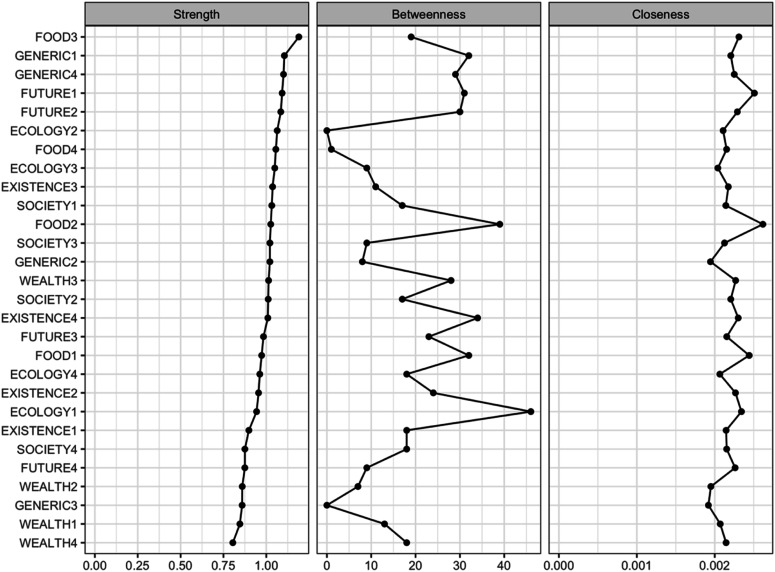
Standardized centrality indices (strength, betweenness, closeness) for the regularized DCCDS network, ordered by strength

The regularized network retained 154 non-zero edges (edge density = .407; Figure 1). The strongest item by Strength centrality was FOOD3 (z = 2.13), followed by GENERIC1 (z = 1.23), GENERIC4 (z = 1.17), and FUTURE1 (z = 1.09; Figure 2). CS-coefficients were .517 for Strength (“good” = CS > .50), .283 for Closeness (“acceptable” = CS > .25), and .206 for Betweenness (below the recommended threshold of .25; 7), indicating that Strength centrality estimates were the most reliable, while Betweenness indices should be interpreted with caution.

**Figure 3.**
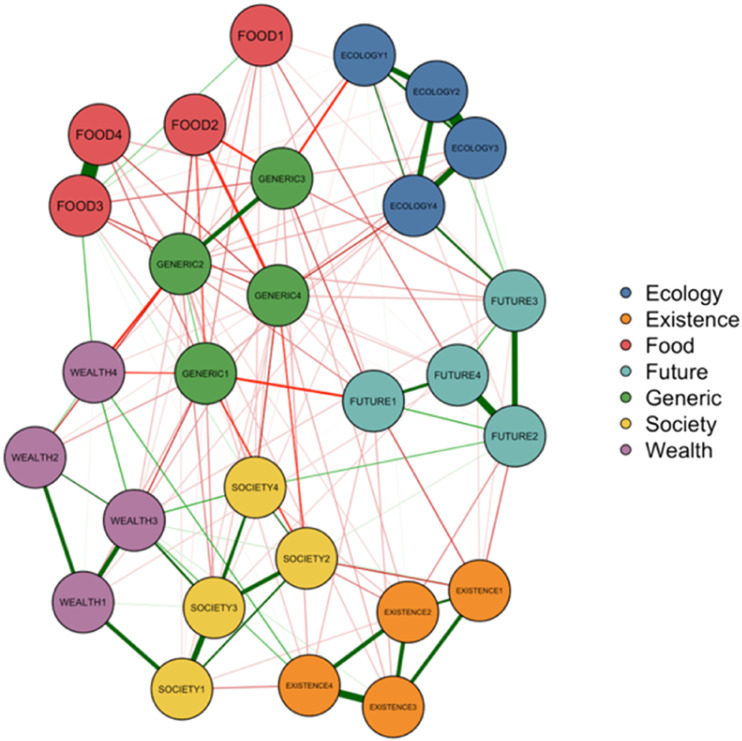
Residual network after partialling out generic factor scores. Green edges indicate positive partial correlations, red edges indicate negative partial correlations, predominantly between generic reference items and domain-specific items

Bridge symptom analysis identified FUTURE1, FOOD2, WEALTH3, FOOD1, and WEALTH4 as the items with the highest bridge strength, reflecting their role as connectors across domain communities. The residual network, estimated after removing generic factor variance, revealed a substantially sparser structure in which domain-specific clusters became clearly visible, with negative inter-cluster partial correlations predominantly involving Generic items (Figure 3). This pattern is consistent with the suppression effects expected in bifactor models.

#### Exploratory Graph Analysis

**Figure 4.**
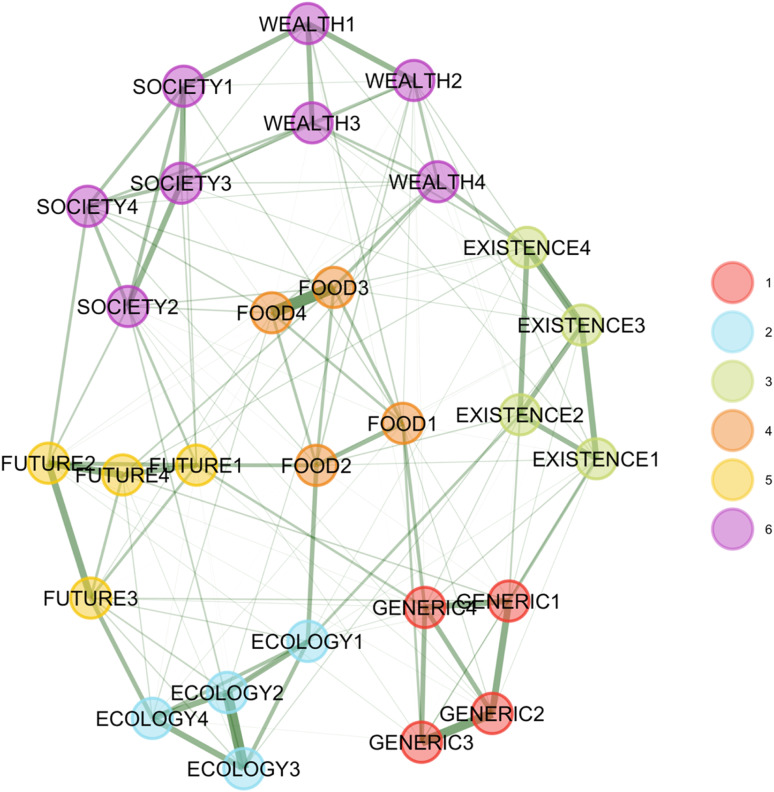
EGA network with empirically detected communities

EGA identified six communities, with all Generic items forming one community, Ecology items a second, Existence items a third, Food items a fourth, Future items a fifth, and Society and Wealth items jointly loading onto a sixth community (Figure 4). Bootstrap EGA (500 replications) yielded a median dimensionality of 6 (95% CI [5.33, 6.67]), with 90.4% of replications supporting a 6-dimensional solution. Item stability coefficients were high throughout (range .75–1.00), with WEALTH4 showing the lowest replication consistency (.75).

### Network Comparison by Gender

The NCT revealed no significant differences in global network structure (p = .264) or global strength (*p* = .782) between men and women, indicating structural equivalence consistent with the measurement invariance findings. At the edge level, 19 of 378 tested edges showed nominal significant gender differences (*p* < .05), a number indistinguishable from the expected false-positive rate of 18.9 edges (378 × .05). After applying a Benjamini-Hochberg false discovery rate correction, none of the 19 edges remained significant (all *p* FDR > .93), confirming that observed edge-level differences reflect chance variation rather than systematic gender differences in network structure.

#### Normative Data

Women reported significantly higher DCCDS scores than men across all domains (all *p*s ⪬ .001), with the largest effects for existential threat (*d* = .58) and generic concerns (*d* = .54). Age-stratified subscale norms showed modest, domain-specific age patterns. Linear age effects were small and, after FDR correction, remained significant only for Generic distress, *b* = −0.158, *p* = .003, *p*_FDR_ = .018, R^2^ = .010, indicating higher distress in younger ages. The linear effect for Existence was nominally significant, *b* = −0.107, *p* = .037, but was not significant after FDR correction, *p*_FDR_ = .130. No significant linear age effects emerged for Ecology, Food, Future, Society, or Wealth, all *p*_FDR_ ≥ .380. Quadratic model comparisons indicated small but significant nonlinear age patterns for Generic distress, R^2^_linear_ = .010, R^2^_quadratic_ = .031, *p*_increment_ < .001; Ecology, R^2^_linear_ = .002, R^2^_quadratic_ = .009, *p*_increment_ = .009; Future, R^2^_linear_ = .002, R^2^_quadratic_ = .026, *p*_increment_ < .001; Society, R^2^_linear_ < .001, R^2^_quadratic_ = .012, *p*_increment_ = .001; and Wealth, R^2^_linear_ < .001, R^2^_quadratic_ = .005, *p*_increment_ = .041. To visualize these nonlinear effects, quadratic age trends were plotted for all domains with significant FDR-corrected quadratic increments (Generic distress, Ecology, Future generations, and Society; see Supplementary Figure S1). Across these domains, scores were comparatively higher among younger adults, decreased toward midlife, and then showed a plateau or slight re-increase at older ages. Quadratic increments were not significant for Existence, R^2^_linear_ = .005, R^2^_quadratic_ = .009, *p*_increment_ = .064, or Food, R^2^_linear_ = .001, R^2^_quadratic_ = .004, *p*_increment_ = .058. Overall, age-related subscale differences were statistically detectable for several domains but explained only a small proportion of variance, supporting the use of age-stratified norms while cautioning against strong substantive interpretation of age effects. For consistency with the FDR-corrected linear terms, the quadratic increments were likewise Benjamini–Hochberg-corrected across the seven domains; after correction, the increment remained significant for Generic, Ecology, Future, and Society (all *p*_FDR_ ≤ .015), whereas the increment for Wealth was no longer significant (*p*_FDR_ = .058), as was also the case for Existence and Food (both *p*_FDR_ = .064). Gender- and age-stratified percentile norms for the DCCDS domain scores are presented in Tables 2 and 3.

**Table 2.** Percentile Norms by Gender

Domain	Gender	n	M (SD)	P25	P50	P75	Cohen’s d
Generic	Men	411	4.06 (1.55)	2.88	4.25	5.25	.54
	Women	474	4.89 (1.48)	4.00	5.00	6.00	
Ecology	Men	411	4.83 (1.55)	3.88	5.00	6.00	.43
	Women	474	5.46 (1.43)	4.75	5.75	6.50	
Existence	Men	411	3.77 (1.49)	2.75	3.75	5.00	.58
	Women	474	4.62 (1.46)	3.75	4.75	5.75	
Food	Men	411	4.71 (1.51)	4.00	5.00	5.75	.31
	Women	474	5.16 (1.42)	4.31	5.50	6.25	
Future	Men	411	4.85 (1.55)	4.00	5.00	6.00	.38
	Women	474	5.42 (1.47)	4.75	5.75	6.50	
Society	Men	411	4.78 (1.43)	3.75	5.00	5.75	.22
	Women	474	5.08 (1.39)	4.25	5.25	6.25	
Wealth	Men	411	4.18 (1.31)	3.38	4.25	5.00	.27
	Women	474	4.52 (1.26)	3.75	4.50	5.25	

*Note.* Items rated on a 7-point scale (1 = not at all to 7 = extremely distressed). Domain scores are means of four items. P25/P50/P75 = 25th/50th/75th percentile. All *p*-values < .001, except Society (*p* = .001). Given the interdependence of subscale scores reflecting a common general factor, a strict Bonferroni correction is overly conservative; nevertheless, all effects remain significant even under this threshold (α = .007), providing robust evidence for the findings.

**Table 3.** Percentile Norms by Age Group

Domain	Age group	n	M (SD)	P25	P50	P75
Generic	<=25	165	5.06 (1.38)	4.25	5.50	6.00
	26-35	238	4.39 (1.46)	3.50	4.50	5.44
	36-45	226	4.28 (1.63)	3.00	4.50	5.50
	46-54	117	4.23 (1.86)	2.50	4.50	5.75
	55+	148	4.59 (1.46)	3.75	4.75	5.75
Ecology	<=25	165	5.40 (1.36)	4.75	5.75	6.50
	26-35	238	5.21 (1.36)	4.50	5.25	6.25
	36-45	226	4.96 (1.66)	3.75	5.25	6.25
	46-54	117	5.03 (1.79)	4.00	5.50	6.50
	55+	148	5.25 (1.45)	4.69	5.50	6.25
Existence	<=25	165	4.47 (1.50)	3.50	4.50	5.50
	26-35	238	4.32 (1.42)	3.31	4.25	5.50
	36-45	226	4.00 (1.56)	3.00	4.00	5.00
	46-54	117	4.10 (1.81)	2.75	4.25	5.50
	55+	148	4.22 (1.44)	3.19	4.25	5.50
Food	<=25	165	5.07 (1.36)	4.25	5.25	6.00
	26-35	238	4.89 (1.35)	4.00	5.00	6.00
	36-45	226	4.81 (1.57)	4.00	5.25	6.00
	46-54	117	4.84 (1.83)	3.50	5.50	6.25
	55+	148	5.19 (1.36)	4.25	5.50	6.00
Future	<=25	165	5.55 (1.33)	4.75	6.00	6.75
	26-35	238	5.12 (1.35)	4.25	5.25	6.19
	36-45	226	4.86 (1.69)	4.00	5.25	6.00
	46-54	117	5.06 (1.91)	3.50	5.75	6.75
	55+	148	5.31 (1.38)	4.50	5.75	6.31
Society	<=25	165	5.23 (1.33)	4.50	5.50	6.25
	26-35	238	4.79 (1.35)	3.81	5.00	5.75
	36-45	226	4.78 (1.46)	4.00	5.00	5.75
	46-54	117	4.93 (1.67)	4.00	5.25	6.25
	55+	148	5.08 (1.31)	4.25	5.38	6.00
Wealth	<=25	165	4.43 (1.19)	3.75	4.50	5.25
	26-35	238	4.41 (1.23)	3.75	4.50	5.25
	36-45	226	4.19 (1.34)	3.50	4.25	5.00
	46-54	117	4.28 (1.59)	3.50	4.50	5.50
	55+	148	4.49 (1.17)	3.75	4.50	5.31

*Note.* Age groups defined by years. P25/P50/P75 = 25th/50th/75th percentile.

## Discussion

The present study provides the most comprehensive psychometric characterization of the DCCDS^5^ to date, drawing on a pooled multi-study sample of N = 894 German-speaking adults.

First, the bifactor S-1 structure replicated robustly, confirming climate distress as a construct with both a strong general dimension and domain-specific facets. The general factor loadings were high and consistent (λ = .544–.841), corroborating the appropriateness of quantifying overall climate distress via a general-factor severity score. At the same time, the specific domains, particularly Ecology, Society and Wealth, carried meaningful residual variance, suggesting that domain-specific scores provide information beyond the general factor. Notably, FOOD1 and FOOD2 showed near-negligible specific loadings, raising the question of whether these items adequately capture food-specific distress or rather contribute primarily to general climate anxiety. Revision of these items in future scale iterations may be warranted. Moreover, several sources may account for the residual misfit. The RMSEA of .079, while within acceptable bounds, is consistent with minor local dependence among semantically overlapping items within domains. The near-zero specific loadings of FOOD1 and FOOD2 indicate that their variance is captured almost entirely by the general factor, adding little domain-specific information and contributing to imperfect fit of the food facet. In addition, the S-1 specification, which constrains the Generic items to load on the general factor only, is deliberately strict, and small unmodeled cross-loadings between conceptually adjacent domains (e.g., Society and Wealth) may further contribute. These considerations point toward targeted item revision rather than a reconsideration of the overall bifactor structure.

Second, measurement invariance analyses demonstrated that the DCCDS measures the same construct in the same way across gender at the structural and loading levels, with only three item intercepts showing meaningful non-invariance. This finding has direct practical implications: researchers can conduct latent mean comparisons between men and women, and the normative data provided here can be applied across gender groups with appropriate caution regarding the three non-invariant items (FOOD1, EXISTENCE1, SOCIETY1). The normative results showed higher DCCDS scores in women than in men, consistent with prior findings that women tend to report higher climate-related anxiety and distress.^3^ Because full metric and partial scalar invariance were supported, these gender comparisons are interpretable, although the freed intercepts, which reflect a small number of items where men and women responded differently regardless of their underlying distress level, should be considered when interpreting item-level differences.

Third, GENERIC1, GENERIC4, FOOD3 and FUTURE1 emerged as statistically central items by Strength, indicating that they are strongly connected within the estimated cross-sectional network. We emphasize, however, that these results do not warrant causal interpretation. The assumption that highly central nodes constitute effective intervention targets has been questioned on both statistical and conceptual grounds.^19-21^ Centrality indices can be unstable, are sensitive to the set of included items, and need not correspond to causal influence. The residual network further revealed that Generic items function as suppressors of domain-specific associations, a pattern theoretically expected under bifactor structures^10^ but rarely visualized in network form.

Fourth, the EGA solution supports six empirical communities rather than the seven theoretical DCCDS domains, because Society and Wealth items clustered together. This finding raises a substantive question about the discriminant validity of these two domains. Future revisions could either merge them into a broader socioeconomic distress domain or sharpen item wording so that Wealth items refer more explicitly to personal financial consequences and Society items to collective and institutional consequences. A central limitation concerns the cultural scope of our data: all seven samples were German-speaking and recruited within a single Western, Central European context. Consequently, the factor structure, the gender invariance, and in particular the normative percentiles reported here cannot be assumed to generalize to non-German-speaking, non-European, or more broadly non-WEIRD populations. Because the salience and endorsement of specific distress domains (e.g., food supply or wealth) are plausibly shaped by national economic conditions and differential exposure to climate impacts, the present results require further testing before the DCCDS and its norms are extended to other linguistic and cultural settings. Additionally, the majority of data were collected during summer months (June/July), which may not be representative of seasonal variation in climate-related distress. Given the temporal clustering of subsamples, potential seasonal or event-driven fluctuations in distress levels cannot be ruled out and warrant investigation in future longitudinal designs.

## Conclusion

The DCCDS demonstrates robust psychometric properties across independent samples and is largely measurement-equivalent across gender. Network analyses identify generic distress, food supply and future generation concerns as the most central distress nodes, but these centrality findings should not be interpreted causally without longitudinal or experimental evidence. The normative data provided here facilitate individual score interpretation in research and applied settings. The empirical evidence for six rather than seven domains invites consideration of a revised domain structure in future iterations of the scale.

## Supplemental Material

Supplemental Material - Psychometric Properties and Network Structure of the Domain-Specific Climate Change Distress Scale Supplemental Material for Psychometric Properties and Network Structure of the Domain-Specific Climate Change Distress Scale by Martin Weiß & Julian Gutzeit in Chronic Stress.

## Data Availability

The anonymized data and analysis code that support the findings of this study are available at https://osf.io/4qmg5/overview.
