# Supplementary material for: Psychometric Properties and Network Structure of the Domain-Specific Climate Change Distress Scale
Source: Chronic Stress (Thousand Oaks). 2026 Jul 18;10:24705470261469261. doi: 10.1177/24705470261469261 (PMC13380697; doi:10.1177/24705470261469261)
Supplement: Supplemental Material - Psychometric Properties and Network Structure of the Domain-Specific Climate Change Distress Scale [file sj-pdf-1-css-10.1177_24705470261469261.pdf]

Supplementary Material

Psychometric Properties and Network Structure of the Domain-Specific Climate Change Distress Scale

Weiß, M. & Gutzeit, J.

Figure S1. Quadratic age trends in DCCDS domain scores. Predicted quadratic age trends are shown for DCCDS domains with significant FDR-corrected quadratic increments: Generic distress, Ecology, Future generations, and Society. Black lines represent predicted scores from quadratic regression models including linear and quadratic age terms. Shaded areas indicate 95% confidence intervals. Small grey points show individual observations. Larger black points indicate empirical mean scores within 10-year age bins, with point size proportional to the number of observations in each bin.

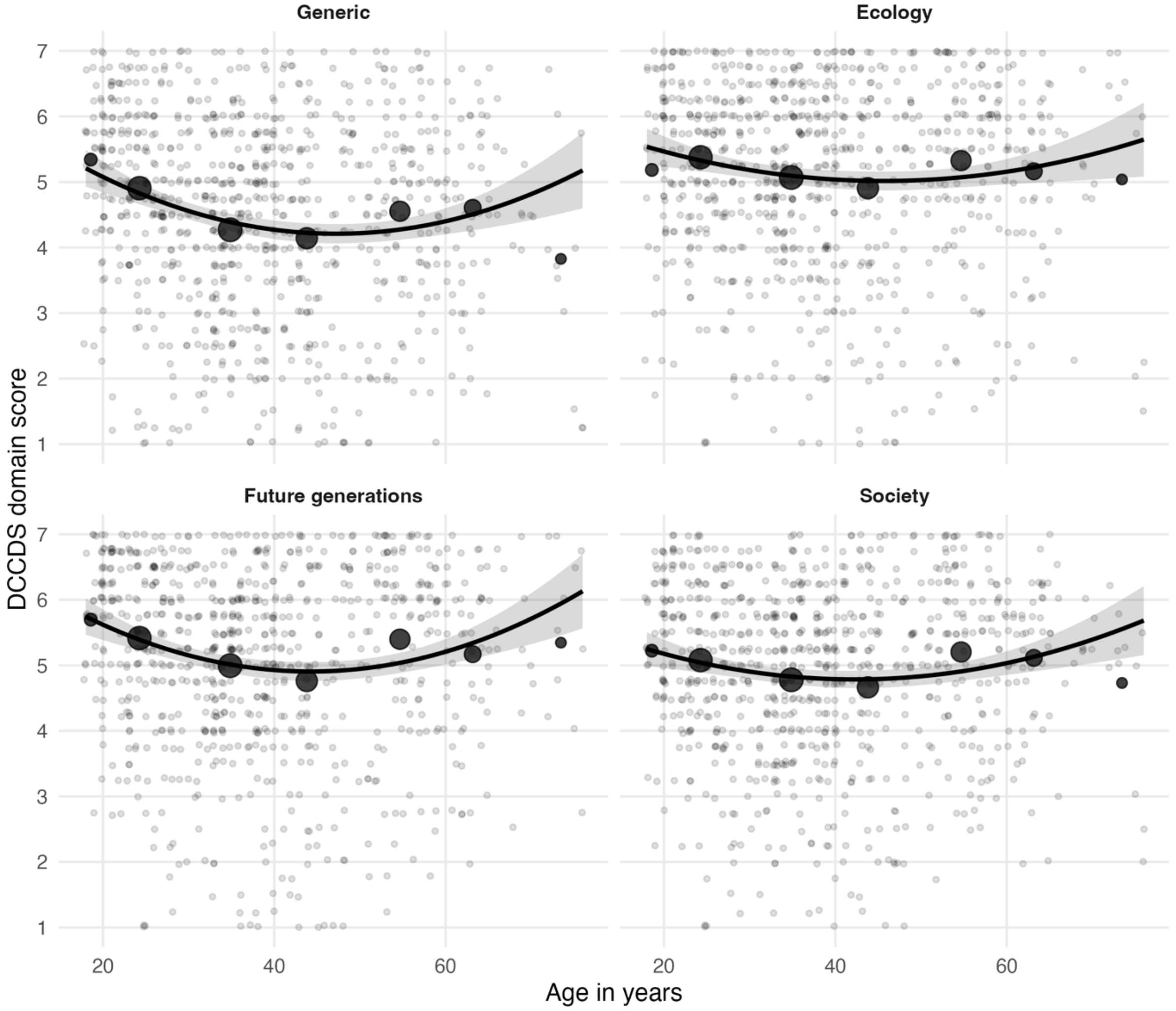

Table S1. Item-Level Descriptive Statistics for All 28 DCCDS Items.

Item	Domain	n	M	SD	Skew	Kurtosis
GENERIC1	Generic	894	4.33	1.70	-0.29	-0.68
GENERIC2	Generic	894	4.46	1.80	-0.36	-0.82
GENERIC3	Generic	894	4.43	1.77	-0.35	-0.82
GENERIC4	Generic	894	4.78	1.73	-0.59	-0.50
ECOLOGY1	Ecology	894	5.03	1.67	-0.78	-0.07
ECOLOGY2	Ecology	894	5.10	1.70	-0.79	-0.16
ECOLOGY3	Ecology	894	5.35	1.61	-1.00	0.33
ECOLOGY4	Ecology	894	5.19	1.68	-0.87	-0.03
EXISTENCE1	Existence	894	3.61	1.86	0.18	-1.01
EXISTENCE2	Existence	894	4.35	1.75	-0.32	-0.77
EXISTENCE3	Existence	894	4.48	1.79	-0.37	-0.84
EXISTENCE4	Existence	894	4.44	1.74	-0.34	-0.76
FOOD1	Food	894	4.83	1.69	-0.62	-0.41
FOOD2	Food	894	4.97	1.63	-0.76	-0.10
FOOD3	Food	894	5.00	1.67	-0.76	-0.19
FOOD4	Food	894	4.99	1.62	-0.75	-0.02
FUTURE1	Future	894	5.09	1.73	-0.80	-0.22
FUTURE2	Future	894	5.40	1.72	-0.98	0.09
FUTURE3	Future	894	5.14	1.70	-0.82	-0.08
FUTURE4	Future	894	5.00	1.77	-0.72	-0.36
SOCIETY1	Society	894	4.81	1.63	-0.57	-0.39
SOCIETY2	Society	894	5.27	1.61	-0.88	0.06
SOCIETY3	Society	894	4.66	1.67	-0.47	-0.54
SOCIETY4	Society	894	5.01	1.71	-0.66	-0.40
WEALTH1	Wealth	894	4.40	1.59	-0.31	-0.52
WEALTH2	Wealth	894	4.15	1.59	-0.25	-0.57
WEALTH3	Wealth	894	3.95	1.59	-0.04	-0.58
WEALTH4	Wealth	894	4.92	1.61	-0.68	-0.10

Note. Items rated on a 7-point Likert scale (1 = not at all distressed to 7 = extremely distressed). M = mean; SD = standard deviation; Skew = skewness; Kurtosis = excess kurtosis. N = 894 for all items (no missing values after listwise deletion).

Table S2. Standardized CFA Factor Loadings from the Bifactor S-1 Model.

	General Factor (K_gen)			Domain-Specific Factors		
Item	λ (std.)	SE	p	λ (std.)	SE	p
GENERIC1	0.806	0.015	< .001			
GENERIC2	0.791	0.018	< .001			
GENERIC3	0.766	0.018	< .001			
GENERIC4	0.841	0.013	< .001			
ECOLOGY1	0.732	0.020	< .001	0.420	0.030	< .001
ECOLOGY2	0.681	0.023	< .001	0.614	0.028	< .001
ECOLOGY3	0.696	0.024	< .001	0.590	0.028	< .001
ECOLOGY4	0.724	0.021	< .001	0.486	0.030	< .001
EXISTENCE1	0.679	0.018	< .001	0.338	0.034	< .001
EXISTENCE2	0.702	0.021	< .001	0.435	0.036	< .001
EXISTENCE3	0.708	0.021	< .001	0.507	0.036	< .001
EXISTENCE4	0.648	0.024	< .001	0.448	0.036	< .001
FOOD1	0.810	0.014	< .001	0.167	0.032	< .001
FOOD2	0.813	0.015	< .001	0.194	0.035	< .001
FOOD3	0.776	0.019	< .001	0.532	0.037	< .001
FOOD4	0.778	0.019	< .001	0.470	0.035	< .001
FUTURE1	0.804	0.016	< .001	0.291	0.037	< .001
FUTURE2	0.738	0.022	< .001	0.483	0.033	< .001
FUTURE3	0.770	0.019	< .001	0.341	0.037	< .001
FUTURE4	0.723	0.021	< .001	0.443	0.037	< .001
SOCIETY1	0.622	0.028	< .001	0.476	0.035	< .001
SOCIETY2	0.726	0.022	< .001	0.409	0.034	< .001
SOCIETY3	0.664	0.025	< .001	0.516	0.036	< .001
SOCIETY4	0.651	0.028	< .001	0.415	0.035	< .001
WEALTH1	0.573	0.030	< .001	0.486	0.042	< .001
WEALTH2	0.652	0.024	< .001	0.407	0.040	< .001
WEALTH3	0.544	0.030	< .001	0.512	0.046	< .001
WEALTH4	0.657	0.028	< .001	0.302	0.043	< .001

Note. Standardized factor loadings (λ) from robust maximum likelihood (MLR) estimation. SE = standard error. All loadings are significant at $p < .001$. The bifactor S-1 model specifies the Generic subscale as the reference factor: Generic items load exclusively on K_gen. Items from all other subscales carry both a K_gen loading and a domain-specific factor loading. Factors are constrained orthogonal to each other.

Table S3. Sample-Wise Demographics.

Sample	Date	N	Age M	Age SD	Range	Men	Women	Other	% women
S1	Jun 2023	305	42.0	12.8	18–76	151	150	4	49.2
S2	Jul 2023	78	41.3	13.6	21–68	27	51	0	65.4
S3	Nov 2023	105	41.8	13.3	18–73	45	59	1	56.2
S4	Jun 2024	145	43.5	12.7	18–76	90	54	1	37.2
S5	Jul 2024	128	33.3	15.1	18–76	50	75	3	58.6
S6	Jun 2025	71	26.3	6.0	19–45	20	51	0	71.8
S7	Feb 2026	62	34.6	4.6	20–40	28	34	0	54.8
Total		894	39.2	13.5	18–76	411	474	9	53.0

Note. Other combines gender codes "diverse" and "prefer not to indicate".

Table S4. Gender-by-Age-Group Cross-Tabulation.

Age group	Men	Women	Total
≤25	51	112	163
26–35	94	144	238
36–45	120	101	221
46–54	60	55	115
55+	86	62	148

Note. The cross-tabulation includes participants coded as men or women only (n = 885).

Table S5. Education and income distribution among available cases.

Education	n	Income	n
No school-leaving qualification	2	No own income	67
Still at school	2	< €250	32
Lower secondary (Hauptschule)	6	€250–<500	40
Intermediate secondary (Mittlere Reife)	60	€500–<1000	85
Completed vocational training	153	€1000–<1500	104
Applied upper secondary (Fachhochschulreife)	71	€1500–<2000	78
Upper secondary (Abitur)	241	€2000–<2500	101
University degree	353	€2500–<3000	70
Other qualification	6	€3000–<3500	48
Missing/not assessed	0	€3500–<4000	24
		€4000 or more	44
		Prefer not to answer	56
		Missing/not assessed	145

Note. Income was assessed in six of the seven samples and available for 749 participants (83.8%); the June 2024 sample (S4, N = 145) did not assess income.

Table S6. Internal Consistency and Model-Based Reliability from the Bifactor S-1 Solution.

Subscale/Scale	M	SD	α	ω/ωs	ωh/ωhs
Total scale	4.76	1.26	.97	.98	.94
Generic	4.50	1.57	.92	.88	—
Ecology	5.17	1.52	.93	.94	.33
Existence	4.22	1.54	.88	.88	.25
Food	4.95	1.49	.92	.93	.14
Future	5.16	1.54	.91	.92	.19
Society	4.94	1.42	.88	.88	.28
Wealth	4.35	1.30	.83	.83	.28

Note. For the total scale, ω = omega total and ωh = omega hierarchical (proportion of total-score variance attributable to the general factor). For subscales, ωs = subscale omega and ωhs = omega hierarchical subscale (specific-factor variance after partialling out the general factor). Generic items define the S-1 reference factor and therefore carry no specific factor.
